# mHealth and Mobile Medical Apps: A Framework to Assess Risk and Promote Safer Use

**DOI:** 10.2196/jmir.3133

**Published:** 2014-09-15

**Authors:** Thomas Lorchan Lewis, Jeremy C Wyatt

**Affiliations:** ^1^Warwick Medical SchoolUniversity of WarwickCoventryUnited Kingdom; ^2^Leeds Institute of Health SciencesFaculty of Medicine, Health & PsychologyUniversity of LeedsLeedsUnited Kingdom

**Keywords:** risk assessment, medical app, mobile health, mobile phone, patient safety, smartphone, mHealth, medical informatics applications

## Abstract

The use of mobile medical apps by clinicians and others has grown considerably since the introduction of mobile phones. Medical apps offer clinicians the ability to access medical knowledge and patient data at the point of care, but several studies have highlighted apps that could compromise patient safety and are potentially dangerous. This article identifies a range of different kinds of risks that medical apps can contribute to and important contextual variables that can modify these risks. We have also developed a simple generic risk framework that app users, developers, and other stakeholders can use to assess the likely risks posed by a specific app in a specific context. This should help app commissioners, developers, and users to manage risks and improve patient safety.

## Introduction

### Overview

The use of mobile medical apps by clinicians, patients, and others has grown dramatically since the introduction of mobile phones and tablet computers. Recent studies show that mobile devices and apps can support a variety of routine medical tasks including clinical reference, drug dose calculation, patient education, accessing medical records, and clinical decision support [1-4]. Mobile phone apps have also been shown to benefit patients in a range of interventions across numerous medical specialties and treatment modalities [5-9]. Medical apps offer clinicians the ability to access medical knowledge and patient data at the point of care with unprecedented ease. However, the intersection of mobile technology, apps, and health care is currently in its most dynamic phase, meaning that there is a need to ensure that patient safety is not compromised before this field matures. For the purposes of this paper, a mobile medical app means any software application created for or used on a mobile device for medical or other health-related purposes. This paper highlights the need for risk assessment to support clinical use of mobile medical apps by critically appraising the existing literature in this field. We identify the different types of risks to which medical apps can contribute and develop a framework that brings together the usage scenarios, contextual factors, and app complexity to estimate the overall probability and severity of harm resulting from use of a mobile medical app.

### Evidence of Unsafe Apps

It is important that mobile medical apps used in health care settings are accurate and reliable, especially as health care professionals and patients may make critical decisions based on information from an app. There is limited literature that addresses the accuracy of mobile medical apps, and that which exists is often highly specialized and not necessarily generalizable to all medical apps [10]. Despite this, several studies have highlighted a number of medical apps that can compromise patient safety and are potentially dangerous in clinical use. For example, certain apps designed for opioid dosage conversion or melanoma detection demonstrate dangerously poor accuracy, while a number of other medical apps do not follow evidence-based guidelines [11-14]. Such risks have led to recent calls for increased regulation before further use and adoption of some apps in clinical practice [15-17]. One issue highlighted by a small number of studies is that many app developers have little or no formal medical training and do not involve clinicians in the development process and may therefore be unaware of patient safety issues raised by inappropriate app content or functioning [18-20]. Another issue is the sheer volume and exponential growth of medical apps, meaning it is practically impossible to assess each and every medical app [21]. The narrow scope of the current evidence base means it is difficult to generalize these statements to all medical and health-related apps. There is sufficient evidence that a small subsection of medical apps presents a risk to patient safety, and therefore it is appropriate to develop a model to help assess these risks.

### Regulatory Oversight

Clinicians trying to safely navigate the apps minefield have had relatively little support from regulatory agencies. The Food and Drug Administration (FDA) released their guidance only in July 2013 after a 2-year consultation period and are focusing primarily on apps that transform the mobile platform into a regulated medical device [22], which to date numbers approximately 100 apps [23]. The remainder will be subject to what the FDA calls “enforcement discretion”, that is, no regulation [24]. Other regulatory agencies such as the Medicines and Healthcare Products Regulatory Agency and the Therapeutic Goods Administration of Australia have offered limited guidance to health care practitioners by including apps under their existing regulations for medical devices [25,26]. The lack of clarity regarding when a medical app becomes a formal medical device means that many developers may not recognize that their app requires formal regulation. As a result, the vast majority of medical apps remain without any form of regulation or safety check, and some of these may present a patient safety or other risk.

## The Need for a Risk Framework to Support Clinical Use of Medical Apps

To inform the safe clinical use of apps and future professional guidance and regulation, it is important to understand and then quantify the different kinds of risk posed by medical apps. It is generally accepted that two dimensions define risk [27]: (1) the probability of an event occurring that could lead to harm, and (2) the severity of the harm that is likely to follow that event.

As with many aspects of medicine, the decision to use a medical app in a particular clinical context relies on our ability to assess the risk of harm and balance it against the anticipated benefits. These judgments require health care professionals to understand the intended benefits, limitations, and risks associated with medical apps in order to make an informed app usage decision. The first step in this process is to identify the different types of risk to which medical apps can contribute, summarized (in broadly increasing order of severity) in Table 1.

There is currently no clinically relevant risk assessment framework for medical apps, so health care practitioners, patients, and app developers find it challenging to quickly assess the risks posed by a specific app. In order to develop a comprehensive risk assessment framework, and to distinguish the different kinds of risk listed in Table 1, we must understand the key variables that can influence risk in medical apps. These variables can be broken down into those risk factors that are inherent to an app and those that depend on the external context where the app is used. Risk factors inherent to an app may be reduced through appropriate regulation, while managing contextual risk factors may require a formal education program to raise awareness among app users. In our opinion, the main contextual and inherent app risk factors are listed in Table 2 below, in no particular order. Arguably many of these risk variables are applicable to many other sources of medical information such as websites or textbooks, although there are important considerations specific to mobile apps that should be recognized.

**Table 1:** Different types of risk that medical use of apps may contribute to, and scenarios where these may arise.

Type of risk in increasing order of severity	Main stakeholder affected	Sample scenario where this risk could arise	What can be done to manage this risk
Loss of reputation	Professional/organization	App displays sensitive performance data about professional or service	Good security
Loss of privacy (patient confidentiality)	Patient	Poor security of patient data	Encryption
		Lose phone holding patient data	Avoid holding patient data on mobile device
Poor quality patient data	Patient/professional/ organization (eg, financial data)	App allows bad data to be entered into patient record or retrieved from it at handover	Data validation on entry and retrieval from authenticated source
Poor lifestyle or clinical decision	Patient/professional	Bad patient data used in risk calculation algorithm	Check correct data retrieved
		Bad knowledge or search tool	Check algorithm properly coded
		Bad advice or algorithm	Use proven health behavior change methods
		Poor risk communication	
Inappropriate but reversible clinical action	Patient/professional	Poor medication advice	Test quality of advice on sample data
			Provide facility for user feedback and respond to this
Inappropriate and irreversible clinical action	Patient/professional/ organization (liability exposure)	Bad algorithm controlling insulin pump, surgical robot, radiotherapy machine, etc	Adopt safety critical software design and development methods
			Exhaustively check design and test algorithm & user interface

**Table 2:** The main inherent and external (contextual) risk variables contributing to the total risk associated with mobile medical apps.

Type of risk variable	Specific risk variable	Explanation
Inherent to the app	Intended function	When the intended function of the app is inherently dangerous, eg, calculating insulin requirements or reprogramming a pacemaker, this will increase risk
	Inaccurate or out of date content	Apps that contain inaccurate or out-of-date content have an increased chance of causing harm
	Complexity of task supported by the app	Apps that carry out complex tasks (eg, drug dosage calculations) have greater potential for harm due to programming errors than simple information display
	Lack of feedback or failsafe mechanism	Apps that do not offer the user a means to report safety issues to the developers are less safe
External factors, depending on context of app use	App user	Use of the app by people other than those intended by the developer may cause harm
	Inappropriate app usage	Apps that are used inappropriately, outside their design envelope, are inherently risky
	Inadequate user training	Even when the app user is as the developer intended, risk can be increased if the user has inadequate training or knowledge to recognize when there is a patient safety hazard, eg, incorrect content or inappropriate advice from the app
	Likelihood of errors being detected	App usage in scenarios with a low error detection capacity (eg, community care versus intensive care) are likely to be riskier
	App usage factor (AUF)	Total number of app users multiplied by the average number of app uses per user per day. Apps with a high usage factor have a greater safety impact on the population than those with a low usage factor

The last two contextual factors are discussed in more detail here. One is the likelihood of a clinical error being detected and averted, which should be high in a well-monitored inpatient or high dependency setting but low when there is only intermittent patient contact, such as in outpatient clinics or primary care. Paradoxically, therefore, the risk of using a faulty app may be lower in an intensive care unit than in general practice. The second is the app usage factor (AUF), which links app risk to the number of users and frequency of use. Risk is proportional to the number of patients affected, so disease prevalence or similar indices of the number of people likely to be affected by an error need to be considered. We have developed the idea of the AUF to help estimate the risk impact of a particular app on a given population. It thus follows that a popular app with a high number of frequent users will have a high AUF and subsequent high impact on the population.

It is also important to consider the generic clinical safety hazards posed by the hardware, software, and sensors that make up a typical medical software application, not just mobile apps. This includes risks posed by the display, user interface, network issues, and subsequent loss of information. Each of these factors should be taken into account, so that the more complex the app, the greater the risk. Unfortunately, these risks are difficult to assess without formal training, but there is guidance for health organizations and developers that aims to address these factors in more detail [28].For the purposes of our risk assessment framework, these factors have been included within the Complexity of task variable.

Developing a formal risk assessment framework for mobile medical apps should enable us to reduce the “residual risk” (exposure to loss remaining after all other known risks have been countered, factored in, or eliminated) by recognizing and implementing a range of possible safety measures in future app development, procurement, and regulation models.

## Bringing Together Usage Scenarios, Contextual Factors, and App Complexity to Estimate Overall Probability and Severity of Harm

We believe that the risks posed by a specific medical app depend on three main dimensions: (1) the probability and the severity of harm, defined by the risk scenarios listed in Table 1, (2) the inherent complexity of the app, which determines how predictable that risk is, and (3) the external or contextual factors listed above.

**Figure 1:**
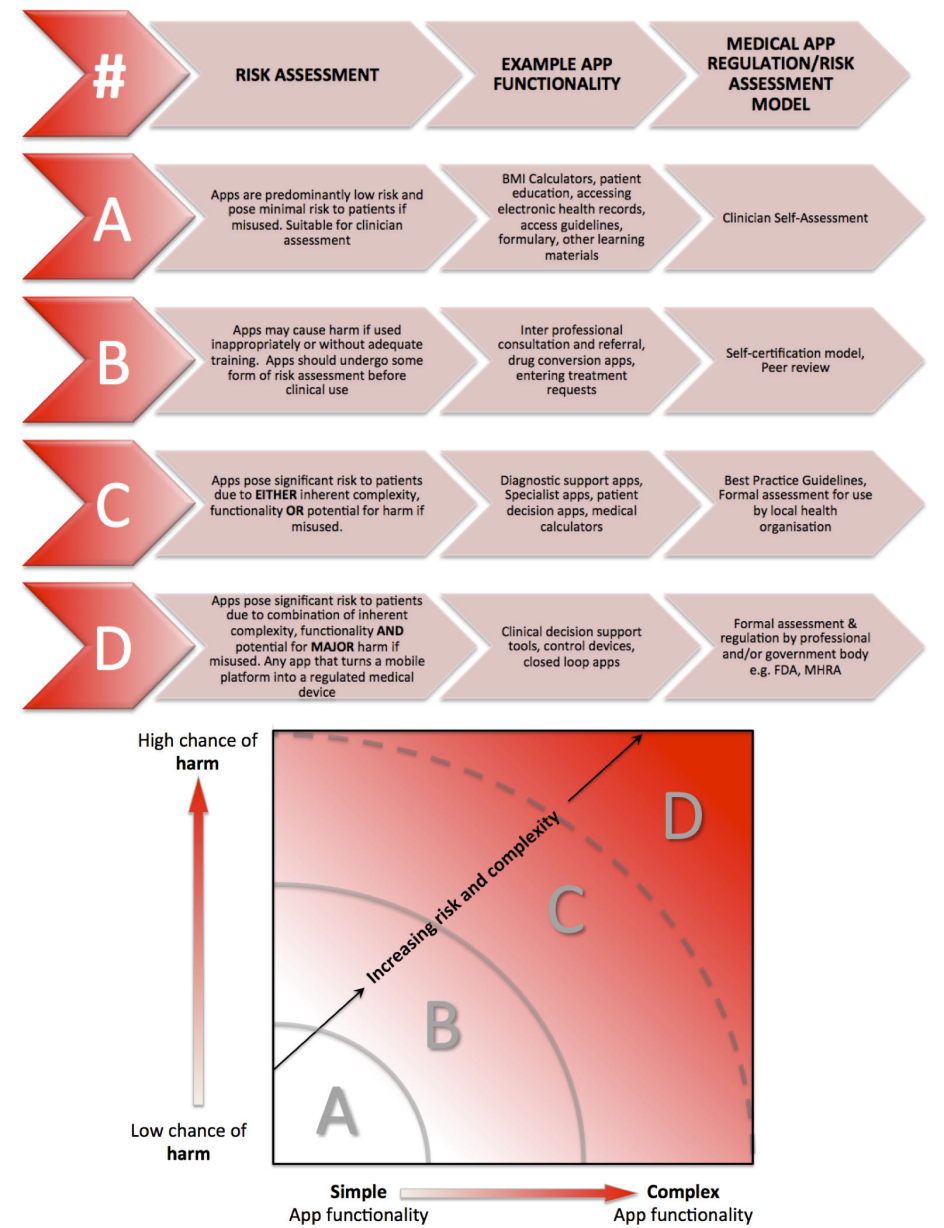
Two-dimensional "App-space" for risk assessment of mobile medical apps with key suggesting appropriate models for app regulation.

Given the wide variety of medical apps, we believe that different approaches to risk assessment and management will be required dependent on app risk. This is illustrated in Figure 1, which shows a 2-dimensional “app-space” where an app can be located depending on its probability of harm, based on the variables above, and its complexity. According to its combined chances of harm and complexity, it will fall into one of four broad zones. Apps in Zone A require only local inspection, those in Zone B require a more formal risk assessment, and those in Zone C require professional review of a full safety case and the use of safety critical development methods. Apps that fall into Zone D should meet the criteria for formal regulation and review by governmental bodies such as the FDA due to their high probability of causing harm. It is not possible to assess the proportion of medical apps in each of the risk categories of A-C given the lack of data on medical apps available. However, based on the total number of medical apps available (approximately 20,000) [29] and the number currently regulated by the FDA (approximately 100) [23], we calculate that the proportion of apps that currently fall into risk category D is approximately 0.5%. This classification into four broad risk zones should help app users, developers, and regulators to evaluate each app using a relevant risk assessment and management model based on the zone where the app is located. It is important to note that these zones form a spectrum rather than discrete entities, hence the gray lines at the boundaries of each zone.

Perhaps the biggest threat to patient safety from medical apps is likely to result from inadequate education and knowledge of health care professionals and patients about their risks. We think in the vast majority of cases, it is probably the actions of a user resulting from a specific app that leads to harm, rather than the app itself. Therefore, an important additional strategy to minimize the risks posed by apps is to develop an educational program to raise awareness of potential patient safety and other risks following inappropriate app use. Developing a single, authoritative, coherent set of guidance and supporting educational materials will require the support of professional bodies such as the Royal Colleges. This will help avoid a confusing plethora of guidance, such as occurred when the harm resulting from some uses of social media was recognized.

In the meantime, there are a range of proposed app regulation models, many of which are highlighted in Figure 1, that may provide some form of protection against hazardous medical apps for patients and health care practitioners [30-33]. Many of these risk management methods are in the early stages of development and have not yet been formally implemented, but they offer a number of advantages for health care professionals, patients, and developers alike, offering some degree of safety check for medical apps not meeting the requirements for formal regulation. A detailed discussion of regulation and regulatory issues for mobile medical apps is beyond the scope of this paper, and interested readers are directed to the references above for further information.

## Conclusions

While the widespread use of high-quality apps by health care practitioners and patients is to be welcomed, there still remains a significant potential for harm. The risks to patient safety and professional reputation are real, and steps should be taken to mitigate these. Identification of all the different kinds of risk and of key variables that influence risk are key stages in the development of a risk assessment model, which should also take into account app complexity and the probability of harm. Education of current health practitioners about the risks posed by medical apps should start soon, before the first case reports of patients harmed by a medical app come to light. Further work should focus on the recognition and mitigation of medical app risk, as the outlook for medical apps in health care is bright once their quality and safety can be reliably assessed and managed.

## Abbreviations

AUF: app usage factor
FDA: Food and Drug Administration
